# Causal Effects and Immune Cell Mediators of Prescription Analgesic Use and Risk of Liver Cancer and Precancerosis in European Population: A Mendelian Randomization Study

**DOI:** 10.3390/biomedicines12071537

**Published:** 2024-07-11

**Authors:** Xuewen Tao, Shuai Mao, Jincheng Wang, Guoqiang Li, Beicheng Sun

**Affiliations:** 1 School of Medicine, Southeast University, Nanjing 210009, China; 18351979632@163.com; 2 Anhui Medical University, Hefei 230022, China; ms19970515@163.com (S.M.); jcwang_med@hotmail.com (J.W.)

**Keywords:** liver cancer and precancerosis, analgesics, immune cell, mendelian randomization

## Abstract

Diverse clinical observations and basic studies have been conducted to explore the implications of analgesic medications in liver diseases. However, the direct causal relationship between prescription analgesic use (PAU) and the risk of liver cancer and precancerosis remains unclear. Thus, we aimed to reveal the conceivable causal effect of PAU on liver cancer and precancerosis, with immune cells as mediating factors. Two-sample Mendelian randomization (MR) analyses were performed to ascertain the causality of PAU on liver cancer and precancerosis. Sensitivity analysis approaches were employed to assess the heterogeneity and pleiotropy of results. Our findings revealed a causal correlation between different PAUs and the risk of liver cancer and alcoholic liver disease (ALD). Specifically, salicylic acid derivatives (SADs) and anilide medications were found to have a protective effect on liver cancer. And non-steroidal anti-inflammatory drugs (NSAIDs) and anilide medications showed a causal impact on ALD. Finally, mediation analyses found that anilide medications influence liver cancer through different immune cell phenotypes. Our research provides new genetic evidence for the causal impact of PAU on liver cancer and precancerosis, with the mediating role of immune cells demonstrated, offering a valuable foundation for researching analgesic medications in liver cancer and precancerosis treatment.

## 1. Background

In Global Cancer Statistics 2020, the epidemiological data for liver cancer revealed that the new incidence rate of liver cancer was approximately 905,677, with 632,320 cases in males and 273,357 in females. Primary liver cancer was also found to be the third leading cause of cancer death worldwide, with 830,180 new deaths in 2020 [1]. The pathogenesis process of liver cancer is multifaceted, involving a variety of molecular mechanisms such as cell cycle dysregulation, chromosomal instability, immune regulation, epithelial–mesenchymal transition, the proliferation of cancer stem cells, and so on [2]. Liver diseases such as liver fibrosis and cirrhosis (LFC) [3], nonalcoholic fatty liver disease (NAFLD) [4], and alcoholic liver disease (ALD) [5] can also progress to liver cancer and are referred to as liver precancerosis. Recent studies have revealed diverse predictive factors associated with the risk of liver cancer, including low-density lipoprotein cholesterol, hypothyroidism, immune cells, and so on [6,7,8]. Although significant advancements have been made, effective systemic therapies for liver cancer are limited. Therefore, it is important to identify new potential predictive factors and develop appropriate medications to cope with the increasing incidence of liver cancer and precancerosis.

In recent years, studies have shown that some analgesics not only have antalgic effects but also have an impact on the growth of tumor cells. For example, morphine is a representative opioid drug, and the correlation between morphine and tumor cell growth has received increasing attention [9,10]. Additionally, studies have shown that long-term use of non-steroidal anti-inflammatory drugs (NSAIDs) is effective in preventing and treating many cancers, including colon cancer [11], liver cancer [12], ovarian cancer, and endometrial cancer [13]. Owing to the increasing analgesic epidemic, opioid medication for chronic noncancer pain led to 168 million opioid prescriptions in the United States in 2018, with prescription pain medications misused by 4% of people aged over 12 [14]. Furthermore, the past 2-week prevalence of nonprescription analgesic medication was 37% in adults in Scotland [15], and 1.3% of patients in general hospitals from a representative sample in Germany were found to have a dependence on analgesics [16]. Although some analgesics have been observed to affect the development of liver cancer [12], the definitive causality and specific mechanism require further study. In addition, whether analgesics affect liver precancerosis is still unclear. Several studies have shown that analgesics such as morphine can affect T cell function [17,18], and T cells also play an important role in the process of liver cancer and precancerosis [19,20,21]. Thus, there may be a potential relationship between prescription analgesic use (PAU) and immune cell-mediated risk of liver cancer and precancerosis.

Mendelian randomization (MR) is an innovative statistical method that employs the natural random distribution of genetic variations in populations to mimic a randomized controlled trial, providing more robust estimates of causality compared to conventional observational studies [22]. By utilizing single-nucleotide polymorphisms (SNPs) as instrumental variables, MR can evaluate causal relationships genetically, minimizing confounding bias and reducing reverse causality risk [23,24]. As immune cells have been found to influence cancer and liver diseases [25,26] and PAU may affect immune cell phenotypes [27], we hypothesized that PAU could decrease the risk of liver cancer and precancerosis through specific immunophenotypes. Thus, this study aimed to investigate the potential causal effects of PAU, including opioids, NSAIDs, salicylic acid derivatives (SAD), anilides, and antimigraine preparations (AP), on the risk of liver cancer, LFC, NAFLD, and ALD, with immune cell phenotypes as mediators.

## 2. Materials and Methods

### 2.1. Study Design

**Figure 1:**
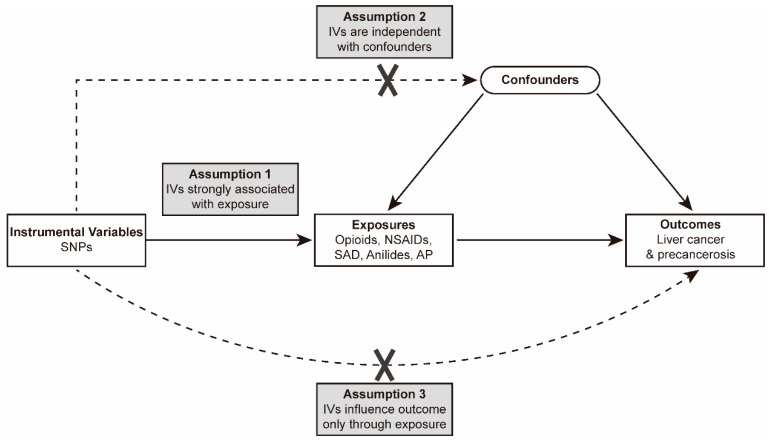
Directed acyclic graph of the MR framework analyzing the causal effect of PAU on liver cancer and precancerosis. Abbreviation: SNPs, single-nucleotide polymorphisms; IVs, instrumental variables; NSAIDs, non-steroidal anti-inflammatory drugs; SAD, salicylic acid derivative; AP, antimigraine preparations.

Our present study applied a two-sample MR method to analyze the potential causality of PAU (including opioids, NSAIDs, SAD, anilides, and AP) on liver cancer and precancerosis (including LCF, NAFLD, and ALD). An MR study uses genetic variations to stand for risk factors, and the valid instrumental variables (IVs) applied for causal inference must meet three main assumptions: 1. Relevance Assumption: the genetic variants are significantly associated with exposures; 2. Independence Assumption: the genetic variants are not associated with any confounders between the exposure and the outcome; 3. Exclusivity Assumption: the genetic variants influence the outcome solely through the exposure, without any other significant associations with the outcome itself (Figure 1) [28]. With SNPs used as IVs for the putative risk factor, MR analyses employ Mendel’s second law of independent genetic inheritance of alleles as a fundamental principle analogous to the random medication treatment in a randomized controlled trial (RCT) [29]. In detail, the research population in the present MR analysis was ‘randomized’ by genetic variants associated with different PAUs. The summary data in our analyses are from pre-approved studies, and no additional ethical approvals or informed consent were required.

### 2.2. Data Sources for Exposures and Outcomes

The open GWAS Catalog (https://gwas.mrcieu.ac.uk/) provided summary-level data for PAU, liver cancer, and precancerosis. To minimize sample overlap in terms of European ancestry, summary statistics for liver cancer and precancerosis were obtained from the FinnGenR9 database (https://r9.finngen.fi/), consisting of up to 377,277 participants: liver cancer with 304 cases and 218,488 controls; LFC with 811 cases and 213,592 controls; NAFLD with 894 cases and 217,898 controls; and ALD with 1416 cases and 217,376 controls. The summary data for liver cancer and LFC in East Asian ancestry were obtained from Biobank Japan. Summary statistics for PAU were derived from a case–control GWAS meta-analysis, including European and East Asian populations [30]. The detailed GWAS Catalog Accession Numbers for the traits of PAU, liver cancer, and precancerosis are exhibited in Supplementary Table S1.

The mediating role of immune cells in the relationship between prescription analgesic use and the risk of liver cancer and precancerosis was explored using publicly available GWAS data for different immune cell signatures, with specific accession numbers ranging from GCST0001391 to GCST0002121 [31]. In total, 731 immunophenotypes were incorporated into the analyses and included four categories: absolute cell counts (*n* = 118), median fluorescence intensities (MFI) reflecting surface antigen levels (*n* = 389), morphological parameters (MP) (*n* = 32), and relative cell counts (*n* = 192).

### 2.3. Selection of Tool Variables

Single nucleotide polymorphisms (SNPs) associated with each PAU and immune cell phenotype trait at the locus-wide significance threshold (*p* < 5 × 10^−8^) were selected. Owing to the limited number of SNPs, the significance level for opioids, liver cancer, or precancerosis trait was set at *p* < 5 × 10^−6^ in accordance with recent studies [32,33]. To minimize bias from linkage disequilibrium (LD) [34], a clumping procedure was processed to prune these SNPs under the following criteria: LD r^2^ threshold < 0.001 and distance = 10,000 kb. The F-statistic was calculated using the following formula: F = R^2^(N − 2)/(1 − R^2^). Here, R^2^ denotes the proportion of exposure variance clarified by the independent variables, N is the effective sample size, and SNPs with an F-statistic < 10 were discarded owing to insufficient strength. With the online tool (https://sb452.shinyapps.io/power/, accessed on 13 May 2024), which was employed to calculate statistical power in MR analyses, the justification for sample sizes for different traits was verified.

### 2.4. Statistical Analysis

All analyses were performed using R 4.3.2 software (http://www.Rproject.org). Several methods were used to evaluate the causal associations between PAU traits and the risk of liver cancer and precancerosis, including inverse variance weighting (IVW) [35], weighted median [36], MR-Egger regression [37], simple mode, and weighted mode [38] using the R packages “TwoSampleMR” (version 0.5.10). All results were expressed as odds ratios (ORs) for binary phenotypes. Cochran’s Q test assessed heterogeneity, and no significant heterogeneity was noted for *p* > 0.05. If heterogeneity was found, fixed-effects IVW was rejected, and random-effect IVW was employed [39]. To avoid horizontal pleiotropy, the MR-Egger intercept [40] and MR pleiotropy residual sum and outlier (MR-PRESSO) [41] method were utilized. 

**Figure 2:**
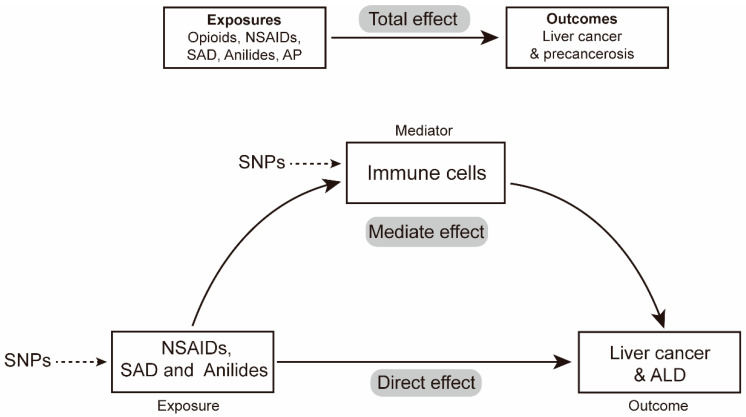
Relationship between analgesic and the risk of liver cancer and precancerosis with immune cells as mediators in the Mendelian randomization. Abbreviations: ALD, alcoholic liver disease.

Based on the causal effects of PAU on the risk of liver cancer and precancerosis (SAD/anilides and liver cancer; NSAIDs/anilides and ALD) revealed in our results, we put forward two further questions that require deeper examination: (1) Is the risk of liver cancer and precancerosis affected by PAU under the mediating role of immune cells? (2) Which immunophenotype is employed by PAU to indirectly regulate the incidence of liver cancer and precancerosis? To investigate these questions, the mediating effect of immune cells was estimated with mediation analysis. This mediation effect estimation was then verified within the MR framework (Figure 2). Generally, the IVW method was employed to estimate the causal effects of NSAIDs, SAD, and anilides on 731 immune cell phenotypes; then, the significant causal effects of immune cells on liver cancer and ALD were analyzed. The total effect of PAU on liver cancer was decomposed into (1) the direct effects of anilides on liver cancer and (2) the indirect effects mediated by anilides with immune cells as mediators. We also calculated the percentage mediated using the mediating effect and the direct effect [42].

## 3. Results

**Figure 3:**
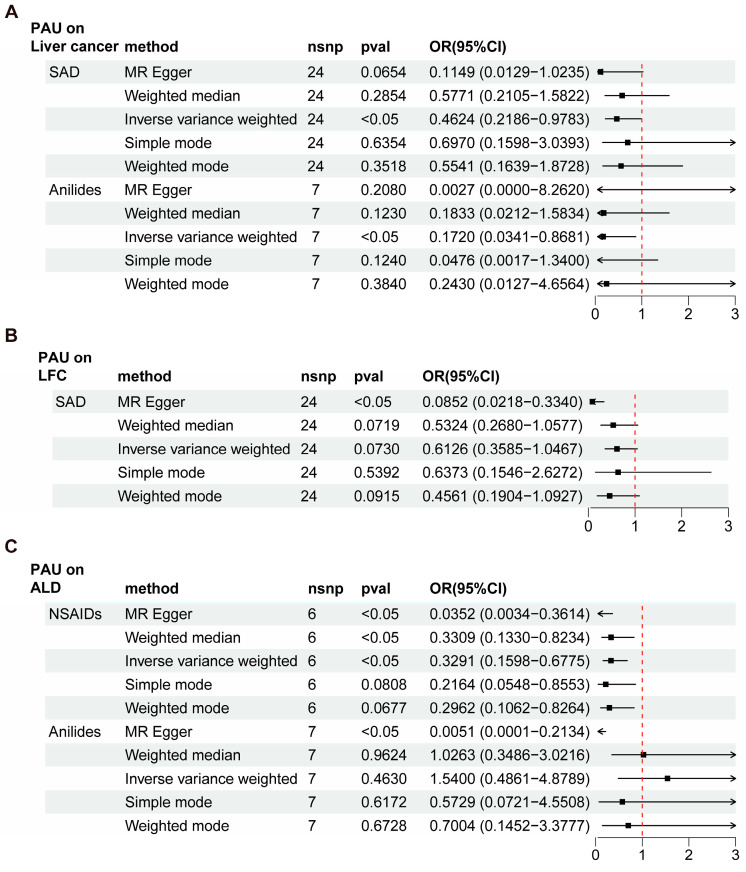
Summary of forest plots showing the significant causal effect of PAU on liver cancer and precancerosis. OR, odds ratio; CI, confidence interval; nsnp, number of single-nucleotide polymorphism; LFC, liver fibrosis and cirrhosis; ALD, alcoholic liver disease.

Based on the above quality control criteria, the IVs significantly associated with different PAUs, namely opioids, NSAIDs, SAD, anilides, and AP, were extracted from the open GWAS Catalog (Supplementary Table S2). Then, we performed two-sample MR analyses to evaluate the causal effects of these five PAU traits on liver cancer, LFC, NAFLD, and ALD, and five of these were statistically significant (Supplementary Figures S1–S4). For liver cancer, we detected the causal effect of two PAU traits (SAD and anilides) (Figure 3A). Specifically, the OR for SAD on liver cancer was evaluated to be 0.4624 (95% CI = 0.2186~0.9783, *p* < 0.05) using the IVW method (Supplementary Table S3). The OR for anilides on liver cancer was evaluated to be 0.1720 (95% CI = 0.0341~0.8681, *p* < 0.05) using the IVW method (Supplementary Table S3). Then, we detected the causal effect of SAD on LFC (Figure 3B). The OR for SAD on LFC was evaluated to be 0.0852 (95% CI = 0.0218~0.3340, *p* < 0.05) using the MR Egger method (Supplementary Table S4). However, a significant causal effect of PAU on NAFLD was not found (Supplementary Figure S3 and Table S5). For ALD, we detected causal effects of two PAU traits (NSAIDs and anilides) (Figure 3C). Specifically, the OR for NSAIDs on ALD was evaluated to be 0.3291 (95% CI = 0.1598~0.6775, *p* < 0.05) using the IVW method. Consistent results were calculated using two additional methods: MR Egger (OR = 0.0352, 95% CI = 0.0034~0.3614) and weighted median (OR = 0.3309, 95% CI = 0.1330~0.88234) (Supplementary Table S6). And the OR for anilides on ALD was evaluated to be 0.0051 (95% CI = 0.0001~0.2134, *p* < 0.05) using the MR Egger method (Supplementary Table S6). These MR estimates suggest a possible protective role of specific PAU in relation to liver cancer and ALD.

Cochran’s Q test was employed for heterogeneity analysis, and the *p*-values of the Q statistics were less than 0.05 in the MR estimates of SAD on LFC and anilides on ALD (Table 1). Thus, a random-effect IVW model was used instead of a fixed-effect model for these two MR estimates. As for the pleiotropy test, the MR-PRESSO global test and MR-Egger intercept ruled out the possibility of horizontal pleiotropy, except for the effect of SAD on LFC (Table 1). Thus, the causal association between SAD and LFC was less convincing.

**Figure 4:**
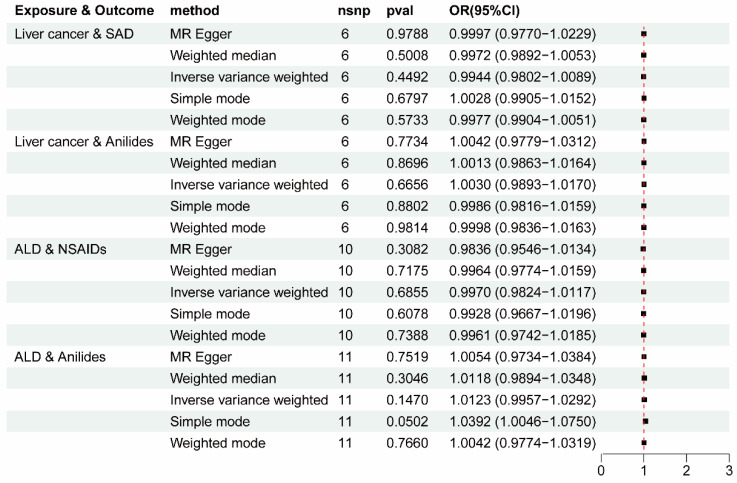
Reverse MR analysis to rule out reverse causation. OR, odds ratio; CI, confidence interval; nsnp, number of single-nucleotide polymorphisms; ALD, alcoholic liver disease.

To eliminate the possibility of potential confounding factors influencing the direction of causal effect, four groups of statistically significant patterns were employed to conduct a reverse MR study, and no significant reverse associations were found (Figure 4 and Supplementary Table S7), laying a foundation for subsequent mediation MR analyses. Then, scatter plots and funnel plots were used to demonstrate the stability of the four statistically significant groups (Supplementary Figures S5 and S6).

**Figure 5:**
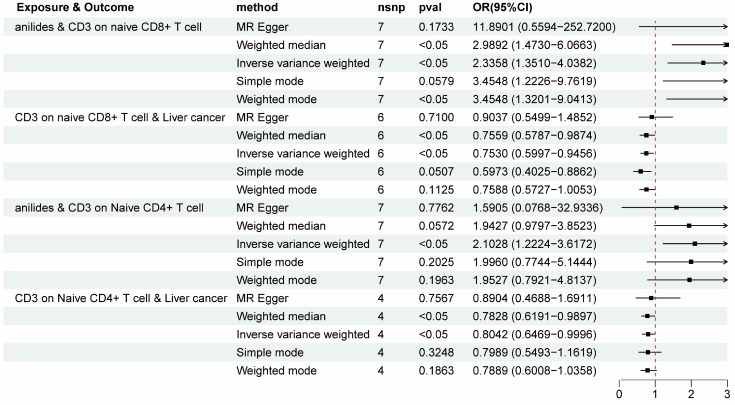
Summary of forest plots showing the significant mediating effect of immune cell phenotypes in the relationship between anilides and liver cancer risk. OR, odds ratio.

It is widely known that immune cells play a crucial role in impacting the processes of liver cancer and ALD. To further enhance our understanding of the effect of various analgesic medications on immune cells and their subsequent impact on liver cancer and ALD, we conducted mediation analyses by screening 731 immune cell phenotypes as mediators. Briefly, IVs at the PAU level were used to estimate the causal effect of exposure on the underlying mediator in the first step. Then, we assessed the causal effect of the immunophenotype mediator on the risk of liver cancer and precancerosis using IVs for the causal mediator-related phenotype [42]. In general, anilide medications were found to be associated with increased CD3 on naive CD8+ T cells (IVW OR = 2.3358, 95% CI = 1.3510~4.0382, *p* < 0.05) and increased CD3 on Naive CD4+ T cells (IVW OR = 2.102778254, 95% CI = 1.2224~3.6172, *p* < 0.05). And protective causality for the impact of CD3 on naive CD8+ T cells (IVW OR = 0.7530, 95% CI = 0.5997 to 0.9456, *p* < 0.05) and CD3 on Naive CD4+ T cells (IVW OR = 0.8041, 95% CI = 0.6469 to 0.9996, *p* < 0.05) on liver cancer were revealed (Figure 5 and Supplementary Table S8). Subsequently, the mediating effect of immune cells in the relationship between anilide drug use and liver cancer risk was revealed. As demonstrated in Table 2, the mediating effect of CD3 on naive CD8+ T cells was −0.2406, and the mediation effect of CD3 on Naive CD4+ T cells was −0.1620, with mediating proportions of 12.5% and 9.2%, respectively (Table 2). These results suggest the protective role of CD3 on naive CD8+ T cells and CD3 on naive CD4+ T cells in mediating the causal association between anilide medications and liver cancer.

**Figure 6:**
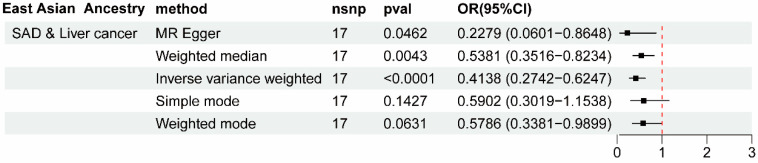
Summary of forest plots showing the significant causal effect of SAD on liver cancer risk in East Asian populations. OR, odds ratio; CI, confidence interval; nsnp, number of single-nucleotide polymorphism.

To further expand the generalizability of our findings to other ancestry groups, we also estimated the causal effects of PAU medications on liver cancer and LFC in East Asian populations. As shown in Figure 6, the drug use of SAD could also significantly decrease liver cancer risk in East Asian ancestry, enhancing the credibility of SAD’s protective effect against liver cancer incidence (Supplementary Table S9). Subsequent heterogeneity and pleiotropy tests supported the robustness of the analysis in East Asian ancestry (Supplementary Figure S7 and Table S10).

## 4. Discussions

Utilizing extensive, publicly accessible genetic databases, our study investigated the causal effect of different PAUs on liver cancer and precancerosis using MR approaches. It is well-known that the progression of liver cancer and precancerosis involves diverse molecular mechanisms. The regenerative capacity of hepatocytes is closely related to the development of liver cancer [43]. And improving the health of sinusoidal endothelial cells can effectively impede NAFLD progression [44], while activation of hepatic stellate cells results in liver fibrosis [45]. Furthermore, immune cells play a pivotal role in liver precancerosis and the transformation to cancer [46]. A recent study explored the causal effect of anti-inflammatory drugs on cancers [47], and our results revealed that genetically predicted SAD and anilide medications were significantly correlated with a decreased incidence of liver cancer. Similarly, the drug use of NSAIDs and anilides might have a significant protective effect on the risk of ALD. 

A recent retrospective cohort study conducted by Dr. Yun et al. demonstrated a significant reduction in the risk of liver cancer among hepatitis B virus-infected persons who used aspirin (hazard ratio = 0.83, 95% CI = 0.75~0.93) [48]. Additionally, a prospective study found that those who take moderate-dose aspirin for 5 years or more had a 59% relative risk reduction in liver cancer compared with people who did not regularly take aspirin [49]. And compatible results were found that long-term use of low-dose aspirin can reduce the risk of liver cancer by 31% and reduce the risk of liver disease-related death by 27% [50]. Mechanistically, aspirin can directly acetylate fibrinogen-like protein 1 and promote its degradation, enhancing immunotherapeutic efficacy and inducing hepatocellular carcinoma regression [51]. Furthermore, aspirin can also trigger ferroptosis in hepatocellular carcinoma cells [52]. Our findings also identified the protective effect of SAD medications on liver cancer in both European and East Asian ancestries, supported by genetic evidence. Thus, the application value of SAD in liver cancer treatment is worthy of serious consideration and research. Although acetaminophen is hepatotoxic, our MR analyses found a protective effect of anilides on the risk of liver cancer. A prospective cohort study revealed that regular paracetamol users had a 28% higher incidence of liver cancer compared to nonusers (HR 1.28, 95% CI 1.06~1.54) [53]. Additionally, Dr. Yang et al. found that the use of paracetamol was associated with a slightly increased risk of liver cancer (multivariable-adjusted OR = 1.18, 95% CI = 1.00~1.39) [12]. Several previous animal studies have also demonstrated the hepatocarcinogenicity of paracetamol [54], so the causal effect of anilides on liver cancer should be interpreted with caution. Further studies focusing on other specific anilides are required to establish causality between anilide medications and liver cancer. 

Our MR analysis also identified NSAIDs and anilides as protective factors in ALD, which has been rarely reported and requires further clinical research. A previous study found that aspirin use was correlated with lower fibrotic indices in suspected ALD [55]. However, researchers have demonstrated that aspirin can suppress the ethanol metabolism process in vivo and potentially increase the adverse effects of alcohol [56]. In addition, a recent study confirmed the function of aspirin and salicylate in inhibiting alcohol dehydrogenase and aldehyde dehydrogenase activities, which are responsible for ethanol metabolism [57]. As researchers have revealed the adverse effects of PAU in ALD, the causality of NSAIDs/anilide medications in ALD requires further basic experiments and clinical trials.

The subsequent two-step MR mediation analyses demonstrated that the protective effect of anilides on liver cancer risk is partially mediated by increased immunophenotypes of CD3 on naive CD8+ T cells and CD3 on naive CD4+ T cells. A recent study demonstrated that, compared to healthy volunteers, hepatocellular carcinoma patients with hepatic B virus have a lower frequency of CD3+ T cells in peripheral blood lymphocytes, indicating the diagnostic role of CD3+ T cells in certain patients with hepatocellular carcinoma [8]. The CD3 complex is a vital signal transduction factor associated with T cell receptors to ensure regulated T cell antigen recognition, signal transduction, and T cell development [58]. CD3-related monoclonal antibodies are mainly used in the clinical treatment of autoimmune-related diseases, and bispecific antibodies involving CD3 have received significant attention as promising tumor therapeutic targets [59]. In addition, the bispecific anti-CD3 antibody catumaxomab has been employed in digestive cancer treatment in a clinical Phase III study. Our analyses also demonstrated the protective role of CD3 on naive CD8+ T cells and CD3 on naive CD4+ T cells in mediating the causality between anilide medications and liver cancer, inspiring the idea of combining anilides with CD3-related immunotherapy for liver cancer treatment. Thus, the hepatotoxicity of anilides may be a main restriction for employing them in liver cancer treatment; thus, developing more specific anilides with attenuated hepatotoxicity is important.

To the best of our knowledge, this is the first MR study designed to explore the mediating role of specific immune cell phenotypes in the causality between genetic susceptibility to different PAUs and the risk of liver cancer and precancerosis. By leveraging genetic instrumental variables and employing various MR methodologies, the causal inference was made while controlling usual confounders, horizontal pleiotropy, and reverse causation. Consistent with previous observational studies, a protective effect of SAD and anilides on liver cancer was identified in this study, providing new genetic evidence for future definitive conclusions. And a novel insight is that immunophenotypes of CD3 on naive CD8+ T cells and CD3 on naive CD4+ T cells were revealed as mediators of the association between anilides and the risk of liver cancer, which may be a potential molecular mechanism for PAU’s protective effect against liver cancer. Recent studies have found that high expression of CD3 represents a protective factor against liver cancer [60], and inhibitory ligands bound to CD3 would impede T cell activation to promote tumor immune evasion [61]. Thus, a combination of specific PAU with CD3-related immunotherapy may be a promising avenue for liver cancer treatment. Additionally, this present MR study demonstrated that NSAIDs and anilide medications can decrease the ALD risk. However, there are currently limited studies exploring the causal association between NSAIDs/anilides and ALD, and further observational prospective studies are needed to verify these results.

Notably, several limitations need to be highlighted in the present study. Firstly, despite conducting multiple sensitivity analyses, horizontal pleiotropy cannot be entirely excluded owing to underlying biological mechanisms. Secondly, as most estimation results of MR analysis were based on a European database, for instance, the causal association between anilides and ALD, the generalizability of these findings is limited to other ethnic groups. Thirdly, MR analyses were not performed for specific drug use; for example, NSAIDs, including ibuprofen and celecoxib, were not individually assessed for their association with ALD risk. This may mask differences in liver cancer and precancerosis risk among different analgesics. Fourthly, the immune system’s complexity suggests that there may be other immune cell phenotypes that act as mediating factors. Finally, drug dosage, frequency of use, and the patient’s specific health status should be taken into account in further randomized case–control trials and cellular biology mechanism research to confirm causality. 

## 5. Conclusions

In summary, we have revealed fresh genetic evidence to support the causal effects of specific PAU on the risk of liver cancer and precancerosis. Several points should be highlighted: (1) SAD was found to decrease the risk of liver cancer in both European and East Asian ancestries. (2) Anilides can decrease the incidence of liver cancer by modulating immune cells. (3) NSAIDs and anilide medications were revealed as protective factors for ALD, which has been rarely reported and should be interpreted with caution. (4) The mediating role of CD3 on naive CD8+ T cells and CD4+ T cells suggests that a combination of specific PAU with CD3 agonist-related immunotherapy may be a promising therapeutic option for liver cancer treatment, and this deserves future study. Overall, these results provide a new theoretical foundation for further research on analgesic medications in the treatment of liver cancer and precancerosis.

## Figures and Tables

**Table 1:** Sensitivity analysis for the causality effects of PAU on liver cancer and precancerosis.

Exposure	Outcome	Heterogeneity Test				Pleiotropy Test		
		Q (MR Egger)	Q_pval (MR Egger)	Q (IVW)	Q_pval (IVW)	Egger Intercept	SE	Global Test *p*
SAD	Liver cancer	25.8421	0.2585	27.9063	0.2193	0.0941	0.0710	0.229
Anilides	Liver cancer	3.1766	0.6728	4.2535	0.6425	0.20484	0.1974	0.082
SAD	LFC	26.7920	0.2192	37.8024	0.0267	0.1335	0.0444	<0.001
NSAIDs	ALD	1.4543	0.8347	5.34232	0.3755	0.1353	0.0686	0.443
Anilides	ALD	4.5954	0.4672	13.9467	0.0302	0.2812	0.0920	0.051

**Table 2:** Mediation effect of analides on the risk of liver cancer via CD3 on naive CD8+ T cells or CD3 on naive CD4+ T cells.

Mediators	Anilides and Liver Cancer						
	Anilides and Mediator		Mediator and Liver Cancer		Direct Effect	Mediation Effect	
	β	*p*	OR	*p*		Effect	Proportion
CD3 on naive CD8+ T cell	0.8483	0.0024	0.7530	0.0146	−1.5403	−0.2406	12.49%
CD3 on naive CD4+ T cell	0.7433	0.0072	0.8042	0.0496	−1.5981	−0.162	9.20%

## Data Availability

Publicly available datasets were analyzed in this study. The details of the repository names and accession numbers can be found in the Supplementary Materials.

## Supplementary Materials

The following supporting information can be downloaded at https://www.mdpi.com/article/10.3390/biomedicines12071537/s1; Supplementary Figures: Scatter plots and funnel plots of different PAU traits on liver cancer and precancerosis. Table S1 (accession numbers): Accession numbers of the GWAS of different traits of PAU, immune cell phenotypes, liver cancer, and precancerosis. Table S2 (selected IVs): Selected SNPs significantly associated with PAU. Tables S3–S6 (analyses results): The analysis results of different MR methods analyzing the causal effect of PAU on liver cancer and precancerosis in European ancestry. Table S7 (Reverse analyses): The analyses results of different MR methods analyzing the causal effect of liver cancer and precancerosis on PAU in European ancestry. Table S8 (Mediating analyses): The analysis results of significant associated immune cells mediating anilides on liver cancer in European ancestry. Table S9 (East Asian MR): The analyses results of different MR methods analyzing the causal effect of SAD on liver cancer in East Asian ancestry. Table S10 (East Asian sensitivity): The sensitivity analysis results of MR estimating the causal effect of SAD on liver cancer in East Asian ancestry.

## Abbreviations

PAU: prescription analgesic use; NSAIDs: non-steroidal anti-inflammatory drugs; SAD: salicylic acid derivatives; AP: antimigraine preparations; MR: Mendelian randomization; GWAS: genome-wide association studies; LFC, Liver fibrosis and cirrhosis; NAFLD, nonalcoholic fatty liver disease; ALD, alcoholic liver disease; SNPs: single-nucleotide polymorphisms; IVs: instrumental variables; ORs: odds ratios; IVW: inverse variance weighting; CI: confidence interval.

## References

[1] Sung H., Ferlay J., Siegel R.L., Laversanne M., Soerjomataram I., Jemal A., Bray F. (2021). Global Cancer Statistics 2020: GLOBOCAN Estimates of Incidence and Mortality Worldwide for 36 Cancers in 185 Countries. CA Cancer J. Clin..

[2] Ogunwobi O.O., Harricharran T., Huaman J., Galuza A., Odumuwagun O., Tan Y., Ma G.X., Nguyen M.T. (2019). Mechanisms of hepatocellular carcinoma progression. World J. Gastroenterol..

[3] Baglieri J., Brenner D.A., Kisseleva T. (2019). The Role of Fibrosis and Liver-Associated Fibroblasts in the Pathogenesis of Hepatocellular Carcinoma. Int. J. Mol. Sci..

[4] Llovet J.M., Willoughby C.E., Singal A.G., Greten T.F., Heikenwälder M., El-Serag H.B., Finn R.S., Friedman S.L. (2023). Nonalcoholic steatohepatitis-related hepatocellular carcinoma: Pathogenesis and treatment. Nat. Rev. Gastroenterol. Hepatol..

[5] Hemminki K., Sundquist K., Sundquist J., Forsti A., Liska V., Hemminki A., Li X. (2023). Personal comorbidities and their subsequent risks for liver, gallbladder and bile duct cancers. Int. J. Cancer.

[6] Cao J., Wang Z., Zhu M., Huang Y., Jin Z., Xiong Z. (2023). Low-density lipoprotein cholesterol and risk of hepatocellular carcinoma: A Mendelian randomization and mediation analysis. Lipids Health Dis..

[7] Lu L., Wan B., Li L., Sun M. (2022). Hypothyroidism has a protective causal association with hepatocellular carcinoma: A two-sample Mendelian randomization study. Front. Endocrinol..

[8] Sun R., Li J., Lin X., Yang Y., Liu B., Lan T., Xiao S., Deng A., Yin Z., Xu Y. (2023). Peripheral immune characteristics of hepatitis B virus-related hepatocellular carcinoma. Front. Immunol..

[9] Gach K., Wyrębska A., Fichna J., Janecka A. (2011). The role of morphine in regulation of cancer cell growth. Naunyn Schmiedebergs Arch. Pharmacol..

[10] Bimonte S., Barbieri A., Palma G., Arra C. (2013). The role of morphine in animal models of human cancer: Does morphine promote or inhibit the tumor growth?. Biomed Res. Int..

[11] Rayyan Y., Williams J., Rigas B. (2002). The role of NSAIDs in the prevention of colon cancer. Cancer Investig..

[12] Yang B., Petrick J.L., Chen J., Hagberg K.W., Sahasrabuddhe V.V., Graubard B.I., Jick S., McGlynn K.A. (2016). Associations of NSAID and paracetamol use with risk of primary liver cancer in the Clinical Practice Research Datalink. Cancer Epidemiol..

[13] Verdoodt F., Kjaer S.K., Friis S. (2017). Influence of aspirin and non-aspirin NSAID use on ovarian and endometrial cancer: Summary of epidemiologic evidence of cancer risk and prognosis. Maturitas.

[14] Keall R., Keall P., Kiani C., Luckett T., McNeill R., Lovell M. (2022). A systematic review of assessment approaches to predict opioid misuse in people with cancer. Support Care Cancer.

[15] Porteous T., Bond C., Hannaford P., Sinclair H. (2005). How and why are non-prescription analgesics used in Scotland?. Fam. Pract..

[16] Fach M., Bischof G., Schmidt C., Rumpf H.J. (2007). Prevalence of dependence on prescription drugs and associated mental disorders in a representative sample of general hospital patients. Gen. Hosp. Psychiatry.

[17] Roy S., Wang J., Charboneau R., Loh H.H., Barke R.A. (2005). Morphine induces CD4+ T cell IL-4 expression through an adenylyl cyclase mechanism independent of the protein kinase A pathway. J. Immunol..

[18] Chandel N., Sharma B., Salhan D., Husain M., Malhotra A., Buch S., Singhal P.C. (2012). Vitamin D receptor activation and downregulation of renin-angiotensin system attenuate morphine-induced T cell apoptosis. Am. J. Physiol. Cell. Physiol..

[19] Hao L., Li S., Hu X. (2023). New insights into T-cell exhaustion in liver cancer: From mechanism to therapy. J. Cancer Res. Clin. Oncol..

[20] Dudek M., Pfister D., Donakonda S., Filpe P., Schneider A., Laschinger M., Hartmann D., Hüser N., Meiser P., Bayerl F. (2021). Auto-aggressive CXCR6(+) CD8 T cells cause liver immune pathology in NASH. Nature.

[21] Lapierre P., Lamarre A. (2015). Regulatory T Cells in Autoimmune and Viral Chronic Hepatitis. J. Immunol. Res..

[22] Davies N.M., Holmes M.V., Davey Smith G. (2018). Reading Mendelian randomisation studies: A guide, glossary, and checklist for clinicians. BMJ.

[23] Lawlor D.A., Harbord R.M., Sterne J.A., Timpson N., Davey Smith G. (2008). Mendelian randomization: Using genes as instruments for making causal inferences in epidemiology. Stat. Med..

[24] Skrivankova V.W., Richmond R.C., Woolf B.A.R., Yarmolinsky J., Davies N.M., Swanson S.A., VanderWeele T.J., Higgins J.P.T., Timpson N.J., Dimou N. (2021). Strengthening the Reporting of Observational Studies in Epidemiology Using Mendelian Randomization: The STROBE-MR Statement. JAMA.

[25] Yin Q., Yang Q., Shi W., Kahlert U.D., Li Z., Lin S., Song Q., Fan W., Wang L., Zhu Y. (2023). Mendelian Randomization Analyses of Chronic Immune-Mediated Diseases, Circulating Inflammatory Biomarkers, and Cytokines in Relation to Liver Cancer. Cancers.

[26] Ming R., Wu H., Liu H., Zhan F., Qiu X., Ji M. (2024). Causal effects and metabolites mediators between immune cell and risk of breast cancer: A Mendelian randomization study. Front. Genet..

[27] Jin Y., Yu X., Li J., Su M., Li X. (2023). Causal effects and immune cell mediators between prescription analgesic use and risk of infectious diseases: A Mendelian randomization study. Front. Immunol..

[28] Boef A.G., Dekkers O.M., le Cessie S. (2015). Mendelian randomization studies: A review of the approaches used and the quality of reporting. Int. J. Epidemiol..

[29] Larsson S.C., Butterworth A.S., Burgess S. (2023). Mendelian randomization for cardiovascular diseases: Principles and applications. Eur. Heart J..

[30] Sakaue S., Kanai M., Tanigawa Y., Karjalainen J., Kurki M., Koshiba S., Narita A., Konuma T., Yamamoto K., Akiyama M. (2021). A cross-population atlas of genetic associations for 220 human phenotypes. Nat. Genet..

[31] Orru V., Steri M., Sidore C., Marongiu M., Serra V., Olla S., Sole G., Lai S., Dei M., Mulas A. (2020). Complex genetic signatures in immune cells underlie autoimmunity and inform therapy. Nat. Genet..

[32] Cao Z., Wu Y., Li Q., Li Y., Wu J. (2022). A causal relationship between childhood obesity and risk of osteoarthritis: Results from a two-sample Mendelian randomization analysis. Ann. Med..

[33] Li W., Wang R., Wang W. (2022). Exploring the causality and pathogenesis of systemic lupus erythematosus in breast cancer based on Mendelian randomization and transcriptome data analyses. Front. Immunol..

[34] Genomes Project C., Auton A., Brooks L.D., Durbin R.M., Garrison E.P., Kang H.M., Korbel J.O., Marchini J.L., McCarthy S., McVean G.A. (2015). A global reference for human genetic variation. Nature.

[35] Hemani G., Zheng J., Elsworth B., Wade K.H., Haberland V., Baird D., Laurin C., Burgess S., Bowden J., Langdon R. (2018). The MR-Base platform supports systematic causal inference across the human phenome. Elife.

[36] Jack B., George D.S., Philip C.H., Stephen B.J.G.E. (2016). Consistent Estimation in Mendelian Randomization with Some Invalid Instruments Using a Weighted Median Estimator. Genet. Epidemiol..

[37] Bowden J., Davey Smith G., Burgess S. (2015). Mendelian randomization with invalid instruments: Effect estimation and bias detection through Egger regression. Int. J. Epidemiol..

[38] Hartwig F.P., Davey Smith G., Bowden J. (2017). Robust inference in summary data Mendelian randomization via the zero modal pleiotropy assumption. Int. J. Epidemiol..

[39] Burgess S., Small D.S., Thompson S.G. (2017). A review of instrumental variable estimators for Mendelian randomization. Stat. Methods Med. Res..

[40] Burgess S., Thompson S.G. (2017). Interpreting findings from Mendelian randomization using the MR-Egger method. Eur. J. Epidemiol..

[41] Verbanck M., Chen C.Y., Neale B., Do R. (2018). Detection of widespread horizontal pleiotropy in causal relationships inferred from Mendelian randomization between complex traits and diseases. Nat. Genet..

[42] Chen Y., Dong H., Qu B., Ma X., Lu L. (2024). Protective effect of higher free thyroxine levels within the reference range on biliary tract cancer risk: A multivariable mendelian randomization and mediation analysis. Front. Endocrinol..

[43] Rigual M.D.M., Sanchez Sanchez P., Djouder N. (2023). Is liver regeneration key in hepatocellular carcinoma development?. Trends Cancer.

[44] Hammoutene A., Rautou P.E. (2019). Role of liver sinusoidal endothelial cells in non-alcoholic fatty liver disease. J. Hepatol..

[45] Wu X., Shu L., Zhang Z., Li J., Zong J., Cheong L.Y., Ye D., Lam K.S.L., Song E., Wang C. (2021). Adipocyte Fatty Acid Binding Protein Promotes the Onset and Progression of Liver Fibrosis via Mediating the Crosstalk between Liver Sinusoidal Endothelial Cells and Hepatic Stellate Cells. Adv. Sci..

[46] Rizvi S., Wang J., El-Khoueiry A.B. (2021). Liver Cancer Immunity. Hepatology.

[47] Gao S., Wei G., Ma Q., Wang X., Wang S., Niu Y. (2024). Causal relationship between anti-inflammatory drugs and cancer: A pan-cancer study with Mendelian randomization. Front. Genet..

[48] Yun B., Ahn S.H., Yoon J.H., Kim B.K. (2022). Clinical Indication of Aspirin Associated with Reduced Risk of Liver Cancer in Chronic Hepatitis B: A Nationwide Cohort Study. Am. J. Gastroenterol..

[49] Simon T.G., Ma Y., Ludvigsson J.F., Chong D.Q., Giovannucci E.L., Fuchs C.S., Meyerhardt J.A., Corey K.E., Chung R.T., Zhang X. (2018). Association Between Aspirin Use and Risk of Hepatocellular Carcinoma. JAMA Oncol..

[50] Simon T.G., Duberg A.S., Aleman S., Chung R.T., Chan A.T., Ludvigsson J.F. (2020). Association of Aspirin with Hepatocellular Carcinoma and Liver-Related Mortality. N. Engl. J. Med..

[51] Lin M., He J., Zhang X., Sun X., Dong W., Zhang R., Xu Y., Lv L. (2023). Targeting fibrinogen-like protein 1 enhances immunotherapy in hepatocellular carcinoma. J. Clin. Investig..

[52] Wang Y.F., Feng J.Y., Zhao L.N., Zhao M., Wei X.F., Geng Y., Yuan H.F., Hou C.Y., Zhang H.H., Wang G.W. (2023). Aspirin triggers ferroptosis in hepatocellular carcinoma cells through restricting NF-kappaB p65-activated SLC7A11 transcription. Acta Pharmacol. Sin..

[53] Tian L., Mi N., Wang L., Huang C., Fu W., Bai M., Gao L., Ma H., Zhang C., Lu Y. (2024). Regular use of paracetamol and risk of liver cancer: A prospective cohort study. BMC Cancer.

[54] Bergman K., Muller L., Teigen S.W. (1996). Series: Current issues in mutagenesis and carcinogenesis, No. 65. The genotoxicity and carcinogenicity of paracetamol: A regulatory (re)view. Mutat Res..

[55] Jiang Z.G., Feldbrugge L., Tapper E.B., Popov Y., Ghaziani T., Afdhal N., Robson S.C., Mukamal K.J. (2016). Aspirin use is associated with lower indices of liver fibrosis among adults in the United States. Aliment. Pharmacol. Ther..

[56] Birkova A., Hubkova B., Cizmarova B., Bolerazska B. (2021). Current View on the Mechanisms of Alcohol-Mediated Toxicity. Int. J. Mol. Sci..

[57] Lee S.L., Lee Y.P., Wu M.L., Chi Y.C., Liu C.M., Lai C.L., Yin S.J. (2015). Inhibition of human alcohol and aldehyde dehydrogenases by aspirin and salicylate: Assessment of the effects on first-pass metabolism of ethanol. Biochem. Pharmacol..

[58] Alcover A., Alarcon B., Di Bartolo V. (2018). Cell Biology of T Cell Receptor Expression and Regulation. Annu. Rev. Immunol..

[59] Deng H., Niu Z., Zhang Z., Zhang J., Wang G., Wang Y., Yang J. (2022). Back on the scene: Advances and challenges in CD3-related drugs in tumor therapy. Drug Discov. Today.

[60] Zheng X., Jin W., Wang S., Ding H. (2021). Progression on the Roles and Mechanisms of Tumor-Infiltrating T Lymphocytes in Patients with Hepatocellular Carcinoma. Front. Immunol..

[61] Deng S., Zhang Y., Wang H., Liang W., Xie L., Li N., Fang Y., Wang Y., Liu J., Chi H. (2024). ITPRIPL1 binds CD3epsilon to impede T cell activation and enable tumor immune evasion. Cell.

