# Supplementary material for: Causal Effects and Immune Cell Mediators of Prescription Analgesic Use and Risk of Liver Cancer and Precancerosis in European Population: A Mendelian Randomization Study
Source: Biomedicines. 2024 Jul 11;12(7):1537. doi: 10.3390/biomedicines12071537 (PMC11274554; doi:10.3390/biomedicines12071537)
Supplement: Supplementary file 1 [file biomedicines-12-01537-s001.zip › Supplementary Figures.pdf]

Supplementary Figures:

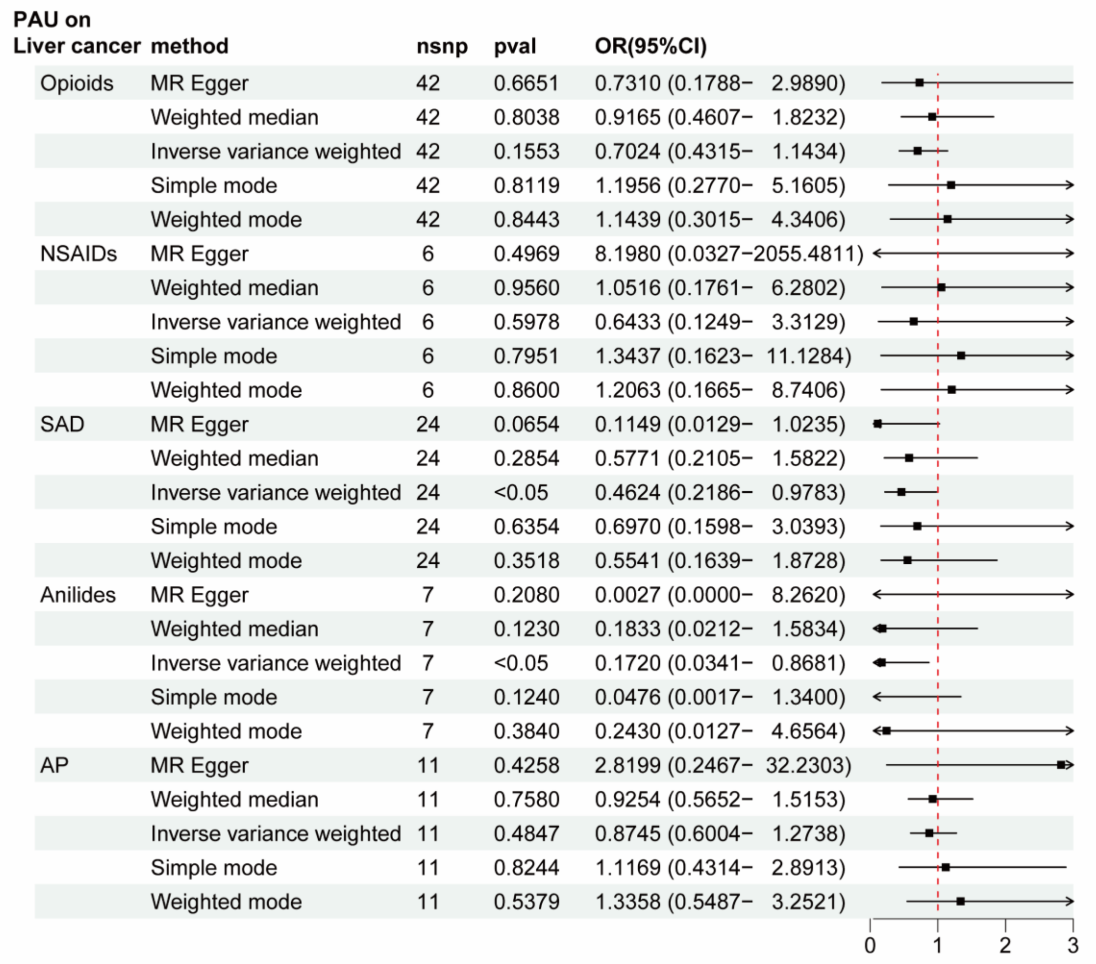

PAU on Liver cancer	method	nsnp	pval	OR(95%CI)
Opioids	MR Egger	42	0.6651	0.7310 (0.1788− 2.9890)
	Weighted median	42	0.8038	0.9165 (0.4607− 1.8232)
	Inverse variance weighted	42	0.1553	0.7024 (0.4315− 1.1434)
	Simple mode	42	0.8119	1.1956 (0.2770− 5.1605)
	Weighted mode	42	0.8443	1.1439 (0.3015− 4.3406)
NSAIDs	MR Egger	6	0.4969	8.1980 (0.0327−2055.4811)
	Weighted median	6	0.9560	1.0516 (0.1761− 6.2802)
	Inverse variance weighted	6	0.5978	0.6433 (0.1249− 3.3129)
	Simple mode	6	0.7951	1.3437 (0.1623− 11.1284)
	Weighted mode	6	0.8600	1.2063 (0.1665− 8.7406)
SAD	MR Egger	24	0.0654	0.1149 (0.0129− 1.0235)
	Weighted median	24	0.2854	0.5771 (0.2105− 1.5822)
	Inverse variance weighted	24	<0.05	0.4624 (0.2186− 0.9783)
	Simple mode	24	0.6354	0.6970 (0.1598− 3.0393)
	Weighted mode	24	0.3518	0.5541 (0.1639− 1.8728)
Anilides	MR Egger	7	0.2080	0.0027 (0.0000− 8.2620)
	Weighted median	7	0.1230	0.1833 (0.0212− 1.5834)
	Inverse variance weighted	7	<0.05	0.1720 (0.0341− 0.8681)
	Simple mode	7	0.1240	0.0476 (0.0017− 1.3400)
	Weighted mode	7	0.3840	0.2430 (0.0127− 4.6564)
AP	MR Egger	11	0.4258	2.8199 (0.2467− 32.2303)
	Weighted median	11	0.7580	0.9254 (0.5652− 1.5153)
	Inverse variance weighted	11	0.4847	0.8745 (0.6004− 1.2738)
	Simple mode	11	0.8244	1.1169 (0.4314− 2.8913)
	Weighted mode	11	0.5379	1.3358 (0.5487− 3.2521)

Supplementary Figure. S1 Summary of forest plots showing significantly causal effect of PAU on liver cancer. OR, odds ratio; CI, confidence interval; nsnp, number of single-nucleotide polymorphis.

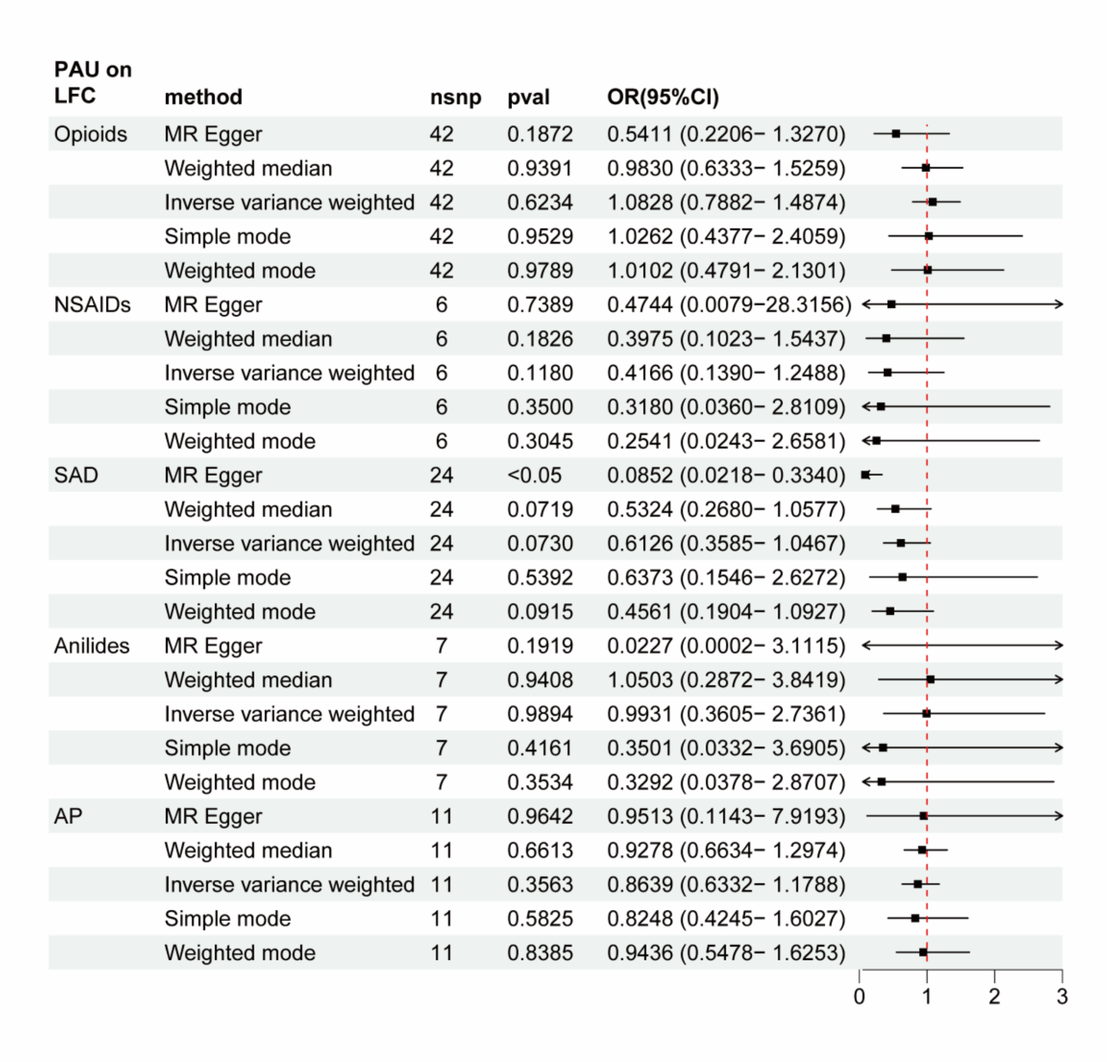

PAU on LFC	method	nsnp	pval	OR(95%CI)
Opioids	MR Egger	42	0.1872	0.5411 (0.2206− 1.3270)
	Weighted median	42	0.9391	0.9830 (0.6333− 1.5259)
	Inverse variance weighted	42	0.6234	1.0828 (0.7882− 1.4874)
	Simple mode	42	0.9529	1.0262 (0.4377− 2.4059)
	Weighted mode	42	0.9789	1.0102 (0.4791− 2.1301)
NSAIDs	MR Egger	6	0.7389	0.4744 (0.0079−28.3156)
	Weighted median	6	0.1826	0.3975 (0.1023− 1.5437)
	Inverse variance weighted	6	0.1180	0.4166 (0.1390− 1.2488)
	Simple mode	6	0.3500	0.3180 (0.0360− 2.8109)
	Weighted mode	6	0.3045	0.2541 (0.0243− 2.6581)
SAD	MR Egger	24	<0.05	0.0852 (0.0218− 0.3340)
	Weighted median	24	0.0719	0.5324 (0.2680− 1.0577)
	Inverse variance weighted	24	0.0730	0.6126 (0.3585− 1.0467)
	Simple mode	24	0.5392	0.6373 (0.1546− 2.6272)
	Weighted mode	24	0.0915	0.4561 (0.1904− 1.0927)
Anilides	MR Egger	7	0.1919	0.0227 (0.0002− 3.1115)
	Weighted median	7	0.9408	1.0503 (0.2872− 3.8419)
	Inverse variance weighted	7	0.9894	0.9931 (0.3605− 2.7361)
	Simple mode	7	0.4161	0.3501 (0.0332− 3.6905)
	Weighted mode	7	0.3534	0.3292 (0.0378− 2.8707)
AP	MR Egger	11	0.9642	0.9513 (0.1143− 7.9193)
	Weighted median	11	0.6613	0.9278 (0.6634− 1.2974)
	Inverse variance weighted	11	0.3563	0.8639 (0.6332− 1.1788)
	Simple mode	11	0.5825	0.8248 (0.4245− 1.6027)
	Weighted mode	11	0.8385	0.9436 (0.5478− 1.6253)

Supplementary Figure. S2 Summary of forest plots showing significantly causal effect of PAU on LFC. OR, odds ratio; CI, confidence interval; nsnp, number of single-nucleotide polymorphism; LFC, Liver fibrosis and cirrhosis.

PAU on NAFLD	method	nsnp	pval	OR(95%CI)
Opioids	MR Egger	42	0.9925	0.9953 (0.3806− 2.6029)
	Weighted median	42	0.6099	0.8941 (0.5815− 1.3746)
	Inverse variance weighted	42	0.7516	0.9481 (0.6815− 1.3189)
	Simple mode	42	0.7426	0.8526 (0.3314− 2.1934)
	Weighted mode	42	0.7040	0.8407 (0.3457− 2.0448)
NSAIDs	MR Egger	6	0.6909	1.8930 (0.1016− 35.2745)
	Weighted median	6	0.2837	1.7970 (0.6154− 5.2473)
	Inverse variance weighted	6	0.0595	2.3222 (0.9667− 5.5786)
	Simple mode	6	0.4626	1.9121 (0.3869− 9.4502)
	Weighted mode	6	0.5984	1.4879 (0.3721− 5.9491)
SAD	MR Egger	24	0.9864	0.9848 (0.1729− 5.6093)
	Weighted median	24	0.6637	1.1373 (0.6367− 2.0315)
	Inverse variance weighted	24	0.6601	1.1376 (0.6404− 2.0210)
	Simple mode	24	0.8743	0.9353 (0.4123− 2.1220)
	Weighted mode	24	0.9783	1.0084 (0.5545− 1.8338)
Anilides	MR Egger	7	0.1384	90.2651 (0.6029−13513.7100)
	Weighted median	7	0.3746	0.5456 (0.1433− 2.0779)
	Inverse variance weighted	7	0.4347	0.6098 (0.1763− 2.1091)
	Simple mode	7	0.4841	0.3968 (0.0349− 4.5066)
	Weighted mode	7	0.5909	0.5078 (0.0489− 5.2734)
AP	MR Egger	11	0.7115	0.7238 (0.1377− 3.8042)
	Weighted median	11	0.3783	1.1555 (0.8378− 1.5937)
	Inverse variance weighted	11	0.3722	1.1190 (0.8742− 1.4322)
	Simple mode	11	0.4543	1.2605 (0.7037− 2.2580)
	Weighted mode	11	0.2790	1.3807 (0.7947− 2.3990)

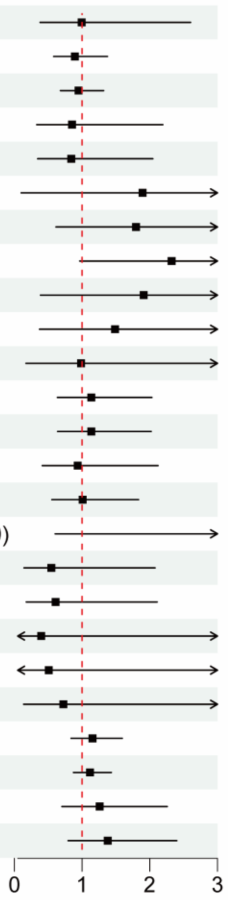

Supplementary Figure. S3 Summary of forest plots showing significantly causal effect of PAU on NAFLD. OR, odds ratio; CI, confidence interval; nsnp, number of single-nucleotide polymorphism; NAFLD, Nonalcoholic fatty liver disease.

PAU on ALD	method	nsnp	pval	OR(95%CI)
Opioids	MR Egger	42	0.2535	0.6441 (0.3060−1.3557)
	Weighted median	42	0.3840	0.8538 (0.5981−1.2188)
	Inverse variance weighted	42	0.6167	0.9363 (0.7236−1.2115)
	Simple mode	42	0.2654	0.6320 (0.2850−1.4017)
	Weighted mode	42	0.2628	0.6539 (0.3140−1.3614)
NSAIDs	MR Egger	6	<0.05	0.0352 (0.0034−0.3614)
	Weighted median	6	<0.05	0.3309 (0.1330−0.8234)
	Inverse variance weighted	6	<0.05	0.3291 (0.1598−0.6775)
	Simple mode	6	0.0808	0.2164 (0.0548−0.8553)
	Weighted mode	6	0.0677	0.2962 (0.1062−0.8264)
SAD	MR Egger	24	0.8709	1.0822 (0.4218−2.7769)
	Weighted median	24	0.9289	0.9791 (0.6154−1.5576)
	Inverse variance weighted	24	0.2751	1.1935 (0.8687−1.6396)
	Simple mode	24	0.1589	1.7591 (0.8225−3.7624)
	Weighted mode	24	0.6658	0.8590 (0.4349−1.6967)
Anilides	MR Egger	7	<0.05	0.0051 (0.0001−0.2134)
	Weighted median	7	0.9624	1.0263 (0.3486−3.0216)
	Inverse variance weighted	7	0.4630	1.5400 (0.4861−4.8789)
	Simple mode	7	0.6172	0.5729 (0.0721−4.5508)
	Weighted mode	7	0.6728	0.7004 (0.1452−3.3777)
AP	MR Egger	11	0.6753	1.3560 (0.3416−5.3826)
	Weighted median	11	0.5333	0.9245 (0.7223−1.1834)
	Inverse variance weighted	11	0.7857	0.9720 (0.7923−1.1925)
	Simple mode	11	0.4354	0.8519 (0.5788−1.2539)
	Weighted mode	11	0.4357	0.8638 (0.6065−1.2301)

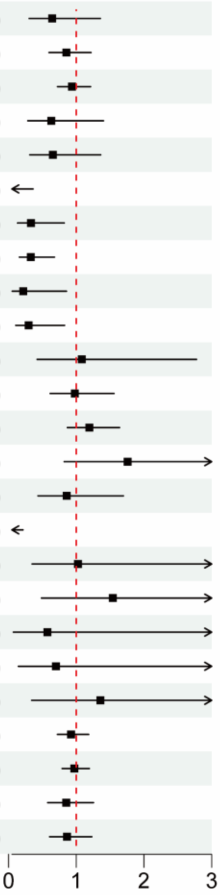

Supplementary Figure. S4 Summary of forest plots showing significantly causal effect of PAU on ALD. OR, odds ratio; CI, confidence interval; nsnp, number of single-nucleotide polymorphism; ALD, Alcoholic liver disease.

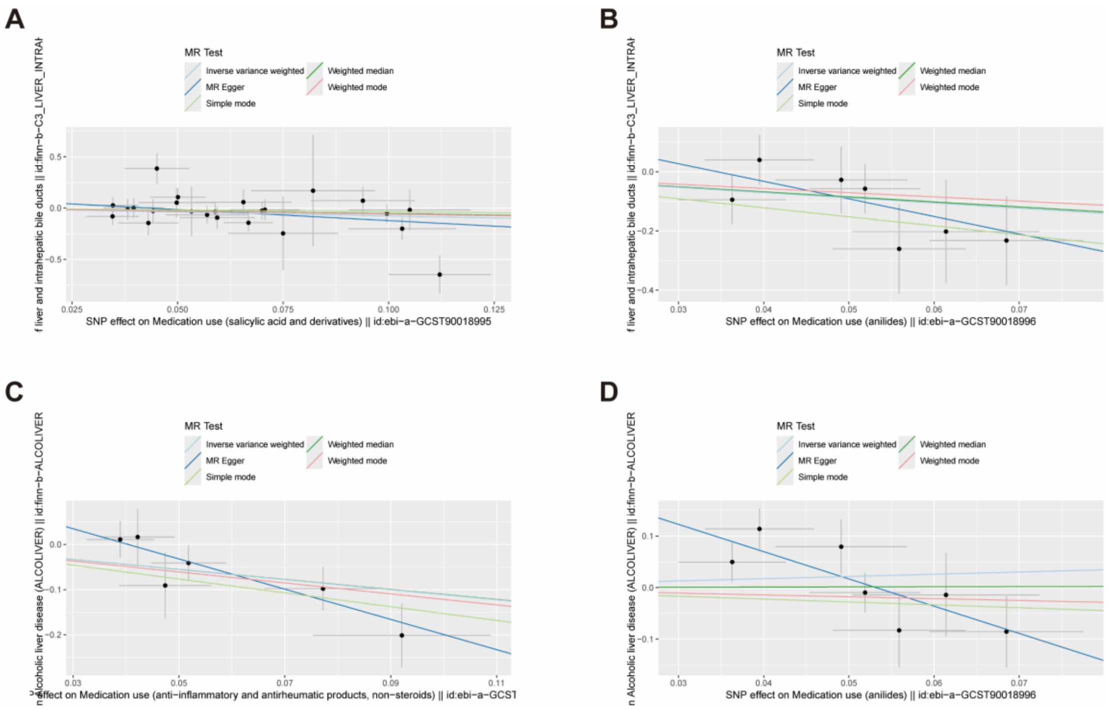

Supplementary Figure. S5 Scatter plots of PAU with the risk of liver cancer and precancerosis. (A) Causal effect of SAD on Liver cancer. (B) Causal effect of Anilides on Liver cancer. (C) Causal effect of NSAIDs on ALD. (D) Causal effect of Anilides on ALD. The effect of the same SNP on exposure is placed on the horizontal axis and the effect on outcome is placed on the vertical axis. The slopes of each line represent the causal association. Abbreviation: PAU: Prescription analgesic use; MR, Mendelian randomization; SNP, single-nucleotide polymorphism; ALD, Alcoholic liver disease.

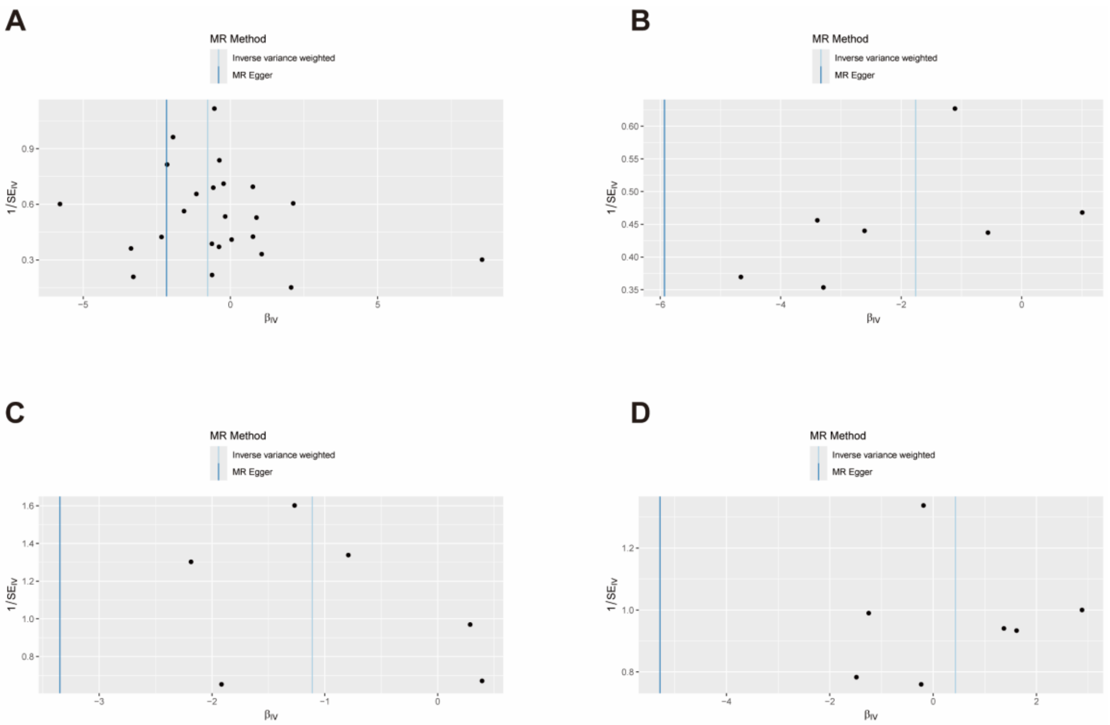

Supplementary Figure. S6 Funnel plots to visualize the overall heterogeneity of MR estimates for the effect of PAU on liver cancer and precancerosis. (A) Causal effect of SAD on Liver cancer. (B) Causal effect of Anilides on Liver cancer. (C) Causal effect of NSAIDs on ALD. (D) Causal effect of Anilides on ALD. The effect of the same SNP on exposure is placed on the horizontal axis and the effect on outcome is placed on the vertical axis. The slopes of each line represent the causal association. Abbreviation: PAU: Prescription analgesic use; MR, Mendelian randomization; SNP, single-nucleotide polymorphism; ALD, Alcoholic liver disease.

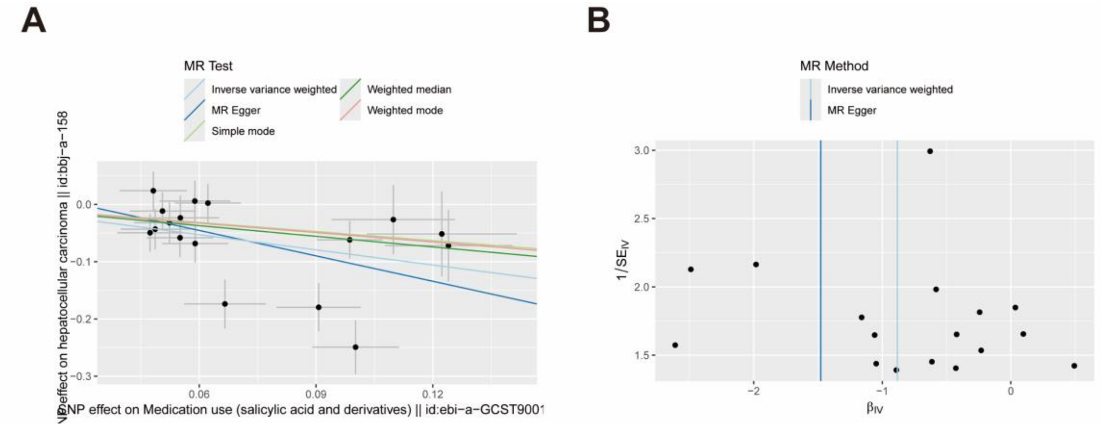

Supplementary Figure. S7 (A) Scatter plots of SAD with the risk of liver cancer in East Asian populations. The effect of the same SNP on exposure is placed on the horizontal axis and the effect on outcome is placed on the vertical axis. The slopes of each line represent the causal association. (B) Funnel plots to visualize the overall heterogeneity of MR estimates for the effect of SAD on liver cancer in East Asian populations. Abbreviation: SAD: salicylic acid derivatives; MR, Mendelian randomization; SNP, single-nucleotide polymorphism;
